# *Toxoplasma gondii* infection of fibroblasts causes the production of exosome-like vesicles containing a unique array of mRNA and miRNA transcripts compared to serum starvation

**DOI:** 10.3402/jev.v2i0.22484

**Published:** 2013-12-11

**Authors:** Samuel M. Pope, Cecilia Lässer

**Affiliations:** 1 Department of Biomedical Sciences, Marian University College of Osteopathic Medicine, Indianapolis IN, USA; 2 Department of Internal Medicine and Clinical Nutrition, Krefting Research Centre, University of Gothenburg, Gothenburg, Sweden

**Keywords:** Toxoplasma, mRNA, miRNA, exosomes, extracellular vesicles, microarray

## Abstract

**Background:**

Until recently thought to be of little significance unless occurring during pregnancy, *Toxoplasma gondii* infection of human hosts is now known to play a larger role in mental health and is a growing concern in the health care community. We sought to elucidate a possible mechanism by which Toxoplasma infection may cause some of the behavioural pathology now associated with infection. We hypothesized that exosomes may be playing a role.

**Methods:**

We utilized electron microscopy to detect the presence and size of extracellular vesicles in the supernatants of Toxoplasma-infected human foreskin fibroblasts (HFF). We then utilized microarray analysis to discern mRNA and miRNA content of the vesicles isolated from supernatants of Toxoplasma-infected (Toxo) and serum-starved (SS) HFF.

**Results:**

We recovered extracellular vesicles with a size consistent with exosomes that we called exosome-like vesicles (ELVs) from the supernatants of SS and Toxo cultures. The mRNA and miRNA content of these ELVs was highly regulated creating specific and unique expression profiles comparing Toxo ELVs, SS ELVs and RNA isolated from whole cell homogenates. Interestingly, among the most enriched mRNA isolated from ELVs of Toxo cells are 4 specific mRNA species that have been described in the literature as having neurologic activity: Rab-13, eukaryotic translation elongation factor 1 alpha 1, thymosin beta 4 and LLP homolog. In addition, miRNA species uniquely expressed in Toxo ELVs include miR-23b, a well-known regulator of IL-17.

**Conclusion:**

While the production of ELVs containing mRNAs that modify behaviour are consistent with reported Toxoplasma pathology, the mechanism of enrichment and ultimate *in vivo* effect of these mRNA and miRNA containing ELVs remains to be investigated.

*Toxoplasma gondii* (Toxoplasma) is a ubiquitous protozoan coccidian parasite of global public health concern. Members of the Felidae (cat) family are the primary hosts, where the parasite undergoes sexual reproduction. All other mammals – including the infection of nearly 2 billion humans worldwide – serve as a secondary host, where the parasite is tropic for the brain. In the brain, the Toxoplasma tachyzoite multiplies via binary fission before entering a metabolically inactive state, bradyzoite, in a cyst-like lesion. This tropism for neurologic tissue is considered by some to be the cause of well-described Toxoplasma-induced behavioural effects in secondary hosts. The cause remains debated; however, recent studies have shown cyst location is random and behavioural effects are not (1, 2). In rodents, Toxoplasma infection causes a reduced fear response to feline odours (3–7). While Toxoplasma infection in humans used to be considered transitory, it is now clear there are long-lasting effects of infection. In humans, Toxoplasma infection is positively correlated with the development of schizophrenia and Alzheimer's disease (8–11). It is known that other protozoans make functional exosomes as reported in the literature for Plasmodium falciparum, Trichomonas sp. and Leishmania (12–14). It has also been reported that Toxoplasma-infected cells make proinflammatory exosomes that increase Tumour necrosis factor-α (TNF-α) production. However, there was no further characterization of those extracellular vesicles (15). It is also known that viral infection causes a cell to change the protein content of the exosomes it secretes (16). Therefore, we hypothesize that Toxoplasma and/or toxoplasma-infected cells produce exosomes that could be a contributing factor to pathology and perhaps behavioural changes in secondary hosts. Exosomes, a sub-population of extracellular vesicles, are small vesicles formed in the endosomal pathway and loaded with various RNAs and proteins. Exosomes are released from the parent cells constitutively, with increases in times of cellular stress. They are then internalized by or bind to neighbouring cells where they release their contents. The contents of the exosomes then exert effects on the recipient cell based on the species of RNA and protein contained in the exosome.

Since the mechanism of Toxoplasma infection associated behavioural pathology is yet to be elucidated, and other protozoa produce exosomes, we hypothesized that exosomes from Toxoplasma or host cells produce exosomes that play a role in pathogenesis. To investigate our hypothesis, we examined supernatants of infected HFF and found extracellular vesicles of the correct size for exosomes that we called exosome-like vesicles (ELVs) by electron microscopy. Furthermore, when we explored the RNA contents using microarrays, we found human mRNA content to be unique when compared to ELVs from uninfected cells.

## Methods

### Cell culture and Toxoplasma infection

Human foreskin fibroblasts (HFF) were grown to confluence in tissue culture DMEM 10% foetal bovine serum (FBS) and infected with approximately 2.0 MOI, multiplicity of infection, of fresh Prugniaud strain of *Toxoplasma gondii* or deprived of FBS. Serum starvation was used as a way to induce exosome production from the HFF at the time points analyzed, as normal levels of exosome production in unstressed HFF did not yield enough RNA for our study. Serum starvation ultimately induces apoptosis but is known to increase the production of extracellular vesicles as well (17). After an incubation period of 24- or 72-hours, the supernatant was collected from the cultures. Each experiment consisted of 20 T-75 flasks per condition that were pooled for vesicles isolation. We conducted 3 experiments at the 72-hour time period and 1 at the 24-hour time period.

### Extracellular vesicle isolation

Extracellular vesicles were isolated by a method outlined in Lässer et al. 2012 (18). In brief, supernatants were harvested from SS and Toxo groups and centrifuged at 300×g for 10 minutes at 4°C. The supernatant was transferred to new centrifuge tubes where it was centrifuged at 16,500×g for 20 minutes at 4°C. The supernatant was then transferred to ultra-centrifuge tubes and centrifuged at 140,000×g for 45 minutes at 4°C. The pellet containing extracellular vesicles was resuspended in 200 µl PBS. A sample was sent for electron microscopy to determine the presence and size of extracellular vesicles.

### RNA isolation

The ELVs-samples were treated with 1,000 U RNAse and DNAse for 20 minutes at 37°C. After nuclease treatment, RNA was purified from the ELVs utilizing the Trizol^©^ RNA purification method as per manufacturer's recommendations. RNA was quantified utilizing UV spectrophotometry.

### RT-PCR

Purified RNA was used to create Template DNA with Invitrogen's First-Strand cDNA Synthesis Kit with Superscript III Reverse Transcriptase^©^ and oligo (dt) as per manufacturer's protocol.

### Microarray procedure

Purified RNA from whole cell lysates, ELVs from Toxoplasma-infected HFF, and ELVs from serum-starved (SS) HFF uninfected with Toxoplasma were submitted for microarray analysis to the University of Pennsylvania Microarray core facility as per their protocols (19). We used the Affymetrix human gene 1.0 st mRNA chip, covering a RefSeq (Entrez) gene count of 21,014 genes, to identify mRNA species in the RNA isolated from our exosome preparations. For microRNA analysis, the Affymetrix microRNA Genechip with Genisphere FlashTag^©^ was used. For Toxoplasma gene product analysis, a proprietary University of Pennsylvania microarray core Toxoplasma expression chip was used. Data analysis providing differential expression values was performed by the University of Pennsylvania's microarray facility utilizing GeneSpring^©^ software. Expression data for mRNA were confirmed by use of RT-PCR amplification of specific mRNA of interest.

### Electron microscopy

Samples were placed as a drop on to Parafilm. Formvar/carbon-coated nickel grids (Ted Pella Inc., Redding, USA), which had been pre-UV-treated for 5 minutes were placed on top of the drop. The samples were fixed in 2% paraformaldehyde and washed with PBS. Next, the samples were immunostained with anti-CD63 antibody (BD Bioscience, Erembodegem, Belgium) or isotype control (Sigma-Aldrich, St. Louis, MO, USA), followed by staining with a 10 nm gold-labelled secondary antibody (Sigma-Aldrich). The samples were then fixed in 2.5% glutaraldehyde, washed in ddH_2_O and contrasted in 2% uranyl acetate. The preparations were examined in a LEO 912AB Omega electron microscope (Carl Zeiss NTS, Jena, Germany).

## Results

**Fig. 1:**
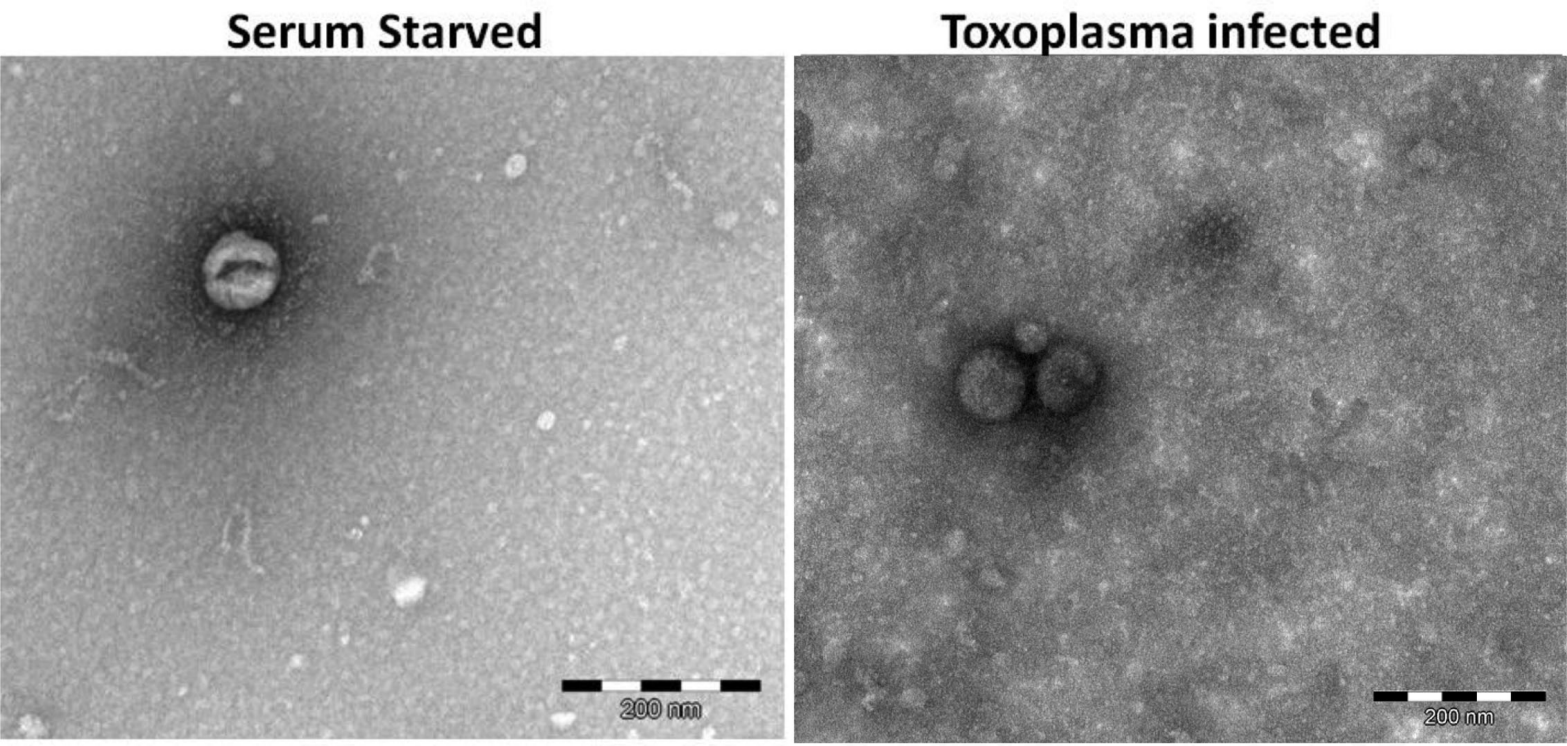
Electron microscopy of vesicle preparations. SS and Toxo vesicle-enriched samples were negative stained and revealed extracellular vesicles of approximately 80–90 nm, the correct dimensions for exosomes.

Electron microscopy revealed extracellular vesicles in the preparations from Toxoplasma-infected HFF (Toxo) and SS HFF, being the correct size of exosomes (<100 nm), but no CD63 was detected (Fig. 1). We believe they are, in fact, exosomes, but we are calling them ELVs until they are further characterized. As our methodology was novel in utilizing microarray analysis of contents of ELVs to examine the effects of Toxoplasma infection on cell culture, it is important to note observations made from initial analysis of the microarray data included as acceptable microarray RNA quality control data (Supplementary File). Furthermore, we observed unique expression profiles for mRNA and miRNA data for ELVs from Toxo HFF compared to ELVs from SS HFF, which differed greatly from samples of ELVs not treated with nuclease and those from whole cell lysates, indicating our methodology of extracellular vesicle purification, RNA isolation and utilization of microarray analysis was successful (Supplementary File). While our methodology was successful and merited further analysis of the mRNA and miRNA data, not all of our data were easily interpreted. We did not see evidence of Toxoplasma RNA in ELVs, but we do not know if that is due to low levels of Toxo gene expression relative to humans or whether they are included at all (Supplementary File). Upon further analysis of the raw comparative human mRNA expression data (Supplementary File), a strategy was developed for picking salient genes from the multitude of those uniquely represented in the ELVs from Toxo compared to SS. First, we ranked the expression of Toxo/SS in descending order revealing 222 mRNA at the 24-hour time point that were more than 5-fold greater in Toxo ELVs compared to SS ELVs. We then screened out RNA with descriptions indicating they were of ribosomal, mitochondrial and microRNA origin, leaving a manageable list of genes that are highly represented in Toxo ELVs compared to SS ELVs. We chose the 11 highest represented mRNA at the 72-hour time point to further investigate (Table I).

**Table I:** mRNA expression in ELVs comparing vesicular mRNA at 24- and 72-hours versus whole cell lysate expression.

	ELVs fold-expression Toxo/SS		Whole cell fold-expression Toxo/SS	
			
mRNA	72 hours	24 hours	24 hours	Identified function
Metallothionein 2A (MT2A)	130.5	47.5	2.4	Frequently observed in invasive human breast cancer (20).
Ferritin, heavy polypeptide 1 (FTH1)	87.0	38.1	−1.2	Iron binding (21).
Eukaryotic translation elongation factor 1 alpha 1 (EEF1A1)	67.6	18.5	−1.2	Multiple functions: role in apoptosis inhibition and linked to autism (22, 23).
Protein transport protein Sec61 subunit gamma (Sec61G)	53.2	21.2	1.1	A subunit of the protein translocation channel at the endoplasmic reticulum (24).
Rab13 (RAS member)	88.6	28.5	1.3	Multiple functions: neuronal regeneration and actin cytoskeletal rearrangements (25, 26).
SET nuclear oncogene (SET)	34.7	9.0	−1.1	Proto-oncogene (27, 28).
Zinc finger protein 253 ( ZNF253)	40.3	29.8	−2.3	Unknown: Assumed DNA binding
RNA, U1 small nuclear 1	33.1	−2.7	1.1	Multiple functionality
Thymosin beta 4, X-linked (TMSB4X)	24.7	4.4	−1.4	Multiple functions: actin sequestration and neuroprotection following traumatic brain injury protection (29–31).
H2A histone family, member Z (H2AFZ)	26.3	11.0	1.4	Histone protein subunit
LLP homolog (LLPH)	5.1	17.8	1.3	Long-term synaptic facilitation (Aplysia) (32, 33).

Values are reported as fold-expression Toxo/SS.

Interestingly, our microarray analysis also highlighted the potential presence of ribosomes in the preparation of our ELVs. It is known that the centrifugal method of ELVs isolation has these issues. We do not feel that this negatively impacts the findings we report today. However, the presence of ribosomes will need to be addressed before investigating function and mechanism of the ELVs. Also of note, it appears that the contents of ELVs are not just a representation of the transcriptome of the cell, as the mRNA species representation ratios, comparing Toxo to SS cells, in ELVs are different from the same comparison using the transcriptome of the whole cell. For example, for ELVs harvested 24 hours post-infection, Metallothionein 2A is represented at 47.49-fold greater concentration in Toxo ELVs than SS ELVs, while whole cell expression of the same gene is only 0.89-fold (Table I). This suggests that Toxoplasma infection and Serum Starvation results in production of unique ELVs and is not just mirroring cell transcription. It is known that Toxoplasma infection has a great influence on cellular transcription (34–36), but this is the first description of the production of specific ELVs contents. It is interesting to note that several of the selected highly represented mRNA in ELVs from Toxoplasma-infected cells have been described in the literature as having neurologic roles. Eukaryotic translation elongation factor 1 alpha 1-autism association, Thymosin beta 4 – provides neuroprotection following traumatic brain injury, LPP-homolog – reported to promote synapse development, and Rab-13 – promotes neurite growth and development (23, 26, 30, 31, 32, 33, 37) . Of the mRNA chosen for closer analysis, several had notable differences between the 24- and 72-hour time point. For example, SET nuclear oncogene, RNA, U1 small nuclear 1 and thymosin beta 4, X-linked (TMSB4X) were all highly represented in the 72-hour time point and less at the 24-hour time point. Also, LLP homolog was more highly represented at the 24-hour time point than at the 72-hour time point.

**Fig. 2:**
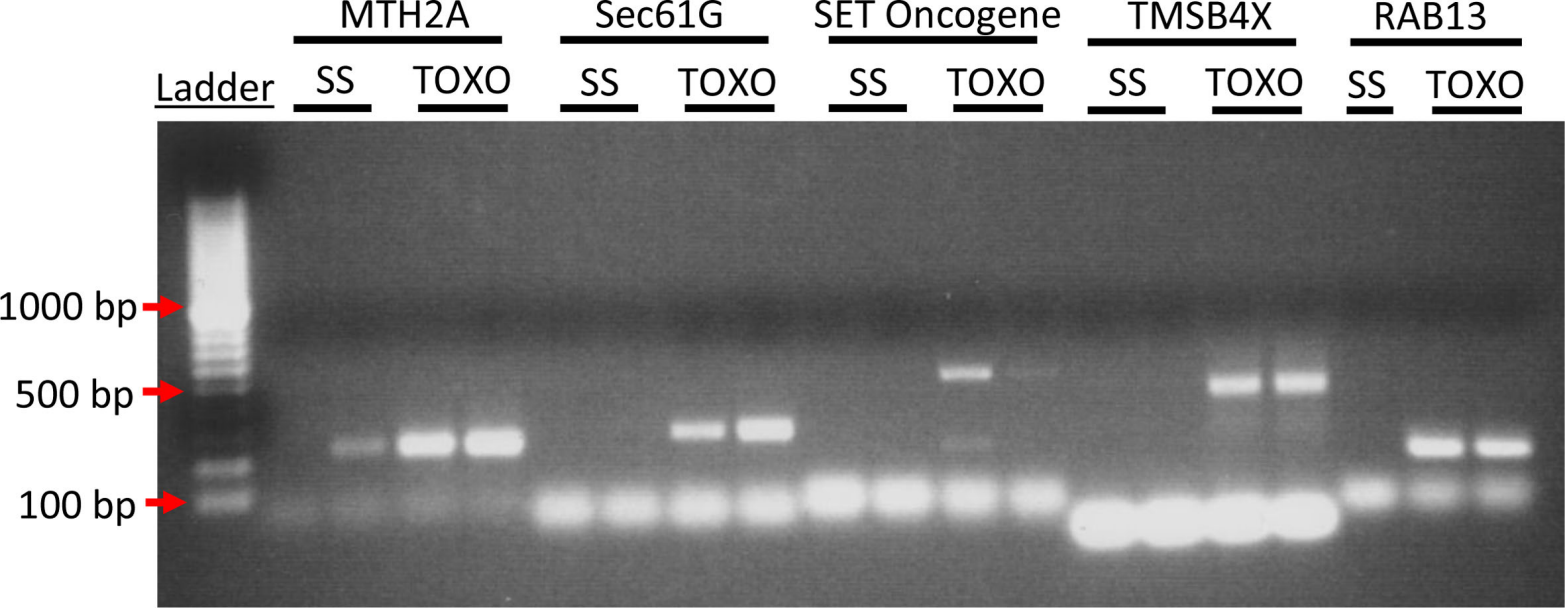
To confirm the microarray data PCR analysis was performed for highly represented genes in Toxo compared to SS ELVs. cDNA from 2 experimental replicates of SS and Toxo ELVs RNA preparations was used to confirm the presence of mRNA transcript for Metallothionein 2A (MTH2A), protein transport protein Sec61 subunit gamma (Sec61G), SET nuclear oncogene (SET), thymosin beta 4, X-linked (TMSB4X), and Rab 13. (Please see Supplementary File for primer sets). The ethidium bromide–treated PCR products were run on a 2% agarose gel and visualized with UV light. Bands of the expected base-pair size are present for these representative mRNA in the Toxoplasma-infected ELVs and are absent from the ELVs of the SS cells except for MTH2A, which shows limited expression in one of the SS samples.

PCR was used to confirm several representative mRNA (Fig. 2). A strong signal was detected within the Toxo ELVs and little to no signal in the SS ELVs for all mRNA primer pairs that produced any signal, including MTH2A, SEC61G, SET Oncogene, TMSB4X and Rab-13. This data strongly confirmed the microarray findings.

**Fig. 3:**
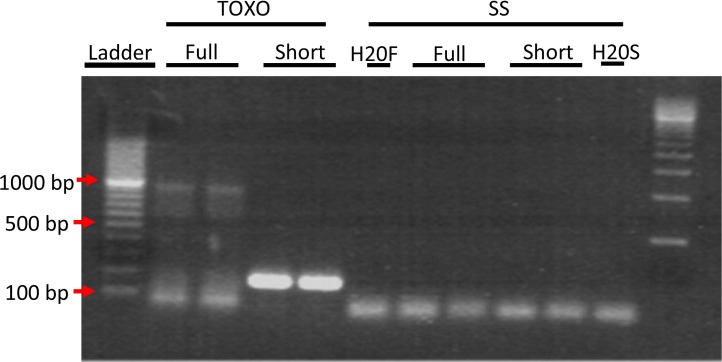
PCR analysis of Rab13 mRNA in Toxo compared to SS ELVs confirmed the presence of full-length transcript in ELVs from TOXO and not in SS ELVs. PCR was performed on cDNA made from SS and Toxo ELVs RNA preparations with primers that were close to full length mRNA and still maintain specificity giving an expected product size of 976 bp. Red arrows indicate location of the 100, 500 and 1,000 bp markers. In lanes 2 and 3 a band of the expected 976 bp size for full length Rab13 can clearly be seen in the ELVs from TOXO and not SS. Also present in lanes 3 and 4, as a positive control, is the 200 bp Rab13 PCR product that was used to confirm the micro array RNA data.

To ensure that full-length mRNA transcript was present in the ELVs and not just fragmented RNA, we performed PCR for the full length Rab13 mRNA. Primers for full length Rab13 mRNA were chosen from NCBI Reference Sequence: NM_002870.2 spanning the mRNA sequence from base pair 111 to 1,086, giving an expected PCR product size of 975 bp. While the PCR reaction was not perfectly clean, showing a smear of smaller products, a distinct band of the expected size was clearly visible just below the 1,000 bp marker (Fig. 3), indicating the presence of full length mRNA in Toxo ELVs.

For miRNA detection, we used the Affymetrix microRNA Genechip^©^ with Genisphere FlashTag^©^. Because of the high homology of miRNA, the chip returned positive expression data in multiple species. We restricted our analysis to human miRNA on the chip (Raw data available in Supplementary File). The high interspecies homology raises the possibility of bovine serum ELVs contamination, which was not an issue with the human mRNA data.

Generally, the enrichment of specific species of miRNA in ELVs was less than that seen in the mRNA data with the greatest difference being 10.8-fold for miRNA compared to 130.5-fold for mRNA. Furthermore, compared to whole cell lysates (Toxo/SS), the contents of ELVs (Toxo/SS) are enriched and not a reflection of cytoplasmic miRNA levels (Table II). A general trend is difficult to ascertain from the miRNA data, because the individual miRNA has been associated with seemingly opposing functions. For example, miR-125b has been reported to cause apoptosis of cancer cells on the one hand and confer a chemoresistant profile on the other (38–40). However, many of the miRNA species detected regulate cell proliferation and are associated with a variety of cancers, including miR-92a, miR-125b, miR-125a-5p, miR-503 and miR-99a-star. Interestingly, one of the most enriched species of miRNA, miR-23b, which is enriched 10.8-fold at the 24-hour time point in Toxo ELVs, plays an anti-inflammatory role causing the down regulation of IL-17 production and response (41).

**Table II:** miRNA expression in ELVs at 24- and 72-hours versus whole cell lysate expression.

	ELVs fold-expression Toxo/SS		Whole cell fold-expression Toxo/SS	
			
miRNA	72 hours	24 hours	24 hours	Identified function
miR-23b	4.6	10.8	−1.1	Cytoskeletal rearrangements and IL-17 regulation (41, 42).
miR-92a	7.5	8.7	1.5	Regulator of apoptosis. Associated with many cancers (43–47).
miR-595	1.3	7.3	−2.2	Upregulated in quiescence and mandibular prognathism (48, 49).
miR-125b	6.5	7.0	−1.4	Regulates cell survival. Associated with many cancers (38, 39, 40, 50) .
miR-199a-3p	3.3	7.0	−1.3	Cytoskeletal remodelling, motility and metastasis (42).
miR-125a-5p	2.3	6.5	−1.4	Activates apoptosis via p53 and other oncogenes (ERBB2) (51, 52).
miR-503	1.3	5.8	−1.2	Inhibits tumour angiogenesis and growth (53).
miR-320d	4.5	5.7	−1.3	Inhibits HSV induced Kaposi sarcoma Herpes Virus (HHV-8) mediated proliferation by inhibiting Replication and Transcription Activator (RTA) (54).
miR-1183	1.9	5.6	1.1	unknown
miR-99a-star	3.2	5.4	1.5	Inhibits cell proliferation (55, 56).

Values are reported as fold-expression Toxo/SS.

## Discussion

The data we present are important because they show that a new mechanism for Toxoplasma pathogenesis is possible. ELVs from infected cells could attach and deliver their contents to uninfected cells, thereby causing the function of uninfected cells to be altered by the addition of the vesicle contents. It is clear from RT-PCR that full-length transcripts of mRNA are present, protected inside of ELVs even after strong nuclease treatment. Full-length mRNA transcripts of neurologically active proteins we report here could alter brain chemistry to the point of behavioural changes. This proposed mechanism would make the exact location of the infected cells less important, as the effect would be spread by the secretion of ELVs from infected cells throughout the brain. If this mechanism is experimentally proven, Toxoplasma could join the ranks of protozoan pathogens that manipulate the host using exosomes, such as *Trichomonas vaginalis* that produces exosomes that deliver their contents to host cells and modulate host cell immune responses and the parasite's adherence to host cells (13); Leishmania sp. producing its own exosomes that influence macrophages to secrete Interleukin-8, necessary to promote infection (14); and *Plasmodium falciparum* which has recently been shown to produce extracellular vesicles to exchange genetic material such as drug resistance genes (12). However, unlike the above listed Protozoans, Toxoplasma does not appear to be releasing ELVs containing its own genetic material.

Here, we have shown that ELVs are produced by Toxoplasma-infected HFF, full length mRNA transcript is present and that ELVs’ RNA contents are not just reflecting a sample of cytoplasmic RNA. Furthermore, RNA from Toxoplasma-infected cell ELVs are distinct from RNA from ELVs produced by SS cells. Indeed, the mRNA microarray data from 24- and 72-hour post-infection time points produced similar results, but not identical, providing further support for the validity of the method and experimental findings. Among the most highly represented mRNA in ELVs from Toxoplasma-infected cells were 4 mRNA for proteins that have been previously reported to have neurologic activity – Eukaryotic translation elongation factor 1 alpha 1, Thymosin beta 4, LPP-homolog and Rab-13. Perhaps adding to the importance of the data is the fact that these ELVs were created by fibroblasts and not neurons, which might be expected to more readily produce significant amounts of neurologically relevant mRNA.

Expression data for miRNA is more difficult to interpret without direct physical experimentation to determine the specific effects, due to many miRNA species having seemingly opposite expression profiles in similar pathologic states: miR-125b reported to cause apoptosis of cancer cells on one hand and conferred a chemoresistant profile to cancer cells on the other (38–40). However, the role of one miRNA seems resolute. miR-23b is an anti-inflammatory mediator that suppresses IL-17 production and response. While it may be intended to ameliorate immune-mediated pathology, suppression of the inflammatory IL-17 response is also likely to aid in the infection process, which would be a similar function reported for Leishmania sp. (14).

The exact impact ELVs from Toxoplasma-infected cells may have on the pathogenesis of Toxoplasma cannot be certain without direct experimentation. However, these data certainly raise the possibility that neurologic effects of Toxoplasma may be, in part, impacted by production of RNA-laden ELVs we described here. It becomes attractive to conjecture that the Toxoplasma parasite could be exerting influence over the exact content of ELVs to promote its own survival. However, much more study needs to be done before such a conclusion could be made. Indeed, little is known about many of the fundamental mechanisms of ELVs production. Among the fundamental questions that beckon for further study are: How is the production of ELVs regulated? How is RNA loaded into ELVs? How does the cell specify which RNA is loaded? Our future studies will focus on these fundamental aspects of ELVs production.
